# Comparison of different second line treatments for metastatic pancreatic cancer: a systematic review and network meta-analysis

**DOI:** 10.1186/s12876-023-02853-w

**Published:** 2023-06-19

**Authors:** Fausto Petrelli, Alessandro Parisi, Gianluca Tomasello, Emanuele Mini, Marcella Arru, Alessandro Russo, Ornella Garrone, Shelize Khakoo, Raffaele Ardito, Michele Ghidini

**Affiliations:** 1 Oncology Unit, ASST Bergamo ovest, Treviglio (BG), 24047 Italy; 2 Clinica Oncologica e Centro Regionale di Genetica Oncologica, Università Politecnica delle Marche, Azienda Ospedaliero-Universitaria delle Marche, Via Conca 71, Ancona, 60126 Italy; 3 Department of Life, Health and Environmental Sciences, University of L’Aquila, L’Aquila, 67100 Italy; 4 Oncology Unit, Fondazione IRCCS Ca’ Granda Ospedale Maggiore Policlinico, Milan, 20122 Italy; 5 General Surgery Unit, ASST Bergamo ovest, Treviglio (BG), 24047 Italy; 6 Department of Medicine, The Royal Marsden Hospital, London, SW3 6JJ UK; 7 Oncological Day Hospital, IRCCS Centro di Riferimento Oncologico Della Basilicata (CROB), Via Padre Pio 1, Rionero in Vulture PZ, 85028 Italy

**Keywords:** Pancreatic adenocarcinoma, Second-line therapy, Chemotherapy, NALIRI, 5-fluorouracil, Gemcitabine

## Abstract

**Background:**

In metastatic pancreatic ductal adenocarcinoma (mPDAC), first line treatment options usually include combination regimens of folinic acid, 5-fluorouracil (5-FU), irinotecan, and oxaliplatin (FOLFIRINOX or mFOLFIRINOX) or gemcitabine based regimens such as in combination with albumin-bound paclitaxel (GEM + nab-PTX). After progression, multiple regimens including NALIRI + 5-FU and folinic acid, FOLFIRINOX, 5-FU-based oxaliplatin doublets (OFF, FOLFOX, or XELOX), or 5-FU-based monotherapy (FL, capecitabine, or S-1) are considered appropriate by major guidelines. This network meta-analysis (NMA) aimed to compare the efficacy of different treatment strategies tested as second-line regimens for patients with mPDAC after first-line gemcitabine-based systemic treatment.

**Methods:**

Randomized phase II and III clinical trials (RCTs) were included if they were published or presented in English. Trials of interest compared two active systemic treatments as second-line regimens until disease progression or unacceptable toxicity. We performed a Bayesian NMA with published hazard ratios (HRs) and 95%confidence intervals (CIs) to evaluate the comparative effectiveness of different second-line therapies for mPDAC. The main outcomes of interest were overall survival (OS) and progression free survival (PFS), secondary endpoints were grade 3–4 toxicities. We calculated the relative ranking of agents for each outcome as their surface under the cumulative ranking (SUCRA). A higher SUCRA score meant a higher ranking for efficacy outcomes.

**Results:**

A NMA of 9 treatments was performed for OS (n = 2521 patients enrolled). Compared with 5-FU + folinic acid both irinotecan or NALIRI + fluoropyrimidines had a trend to better OS (HR = 0.76, 95%CI 0.21–2.75 and HR = 0.74, 95%CI 0.31–1.85). Fluoropyrimidines + folinic acid + oxaliplatin were no better than the combination without oxaliplatin. The analysis of treatment ranking showed that the combination of NALIRI + 5-FU + folinic acid was most likely to yield the highest OS results (SUCRA = 0.7). Furthermore, the NMA results indicated that with the highest SUCRA score (SUCRA = 0.91), NALIRI + 5-FU + folinic acid may be the optimal choice for improved PFS amongst all regimens studied.

**Conclusions:**

According to the NMA results, NALIRI + 5-FU, and folinic acid may represent the best second-line treatment for improved survival outcomes in mPDAC. Further evidence from prospective trials is needed to determine the best treatment option for this group of patients.

**Supplementary Information:**

The online version contains supplementary material available at 10.1186/s12876-023-02853-w.

## Introduction

Pancreatic ductal adenocarcinoma (PDAC) is one of the deadliest malignancies worldwide [1]. Radical resection with curative intent can only be performed in < 15% of patients with localized tumors. Chemotherapy is the recommended initial treatment modality in the borderline resectable, locally advanced or metastatic setting with subsequent chemo-radiotherapy being considered for non-metastatic cases that remain unresectable. In the metastatic disease setting, in patients with good performance status, the combination regimen of folinic acid, 5-fluorouracil (5-FU), irinotecan, and oxaliplatin (FOLFIRINOX or mFOLFIRINOX) showed significant progression-free survival (PFS) and overall survival (OS) benefit compared to gemcitabine alone but at the cost of higher toxicity rates. Additionally, the combination of gemcitabine plus albumin-bound paclitaxel (GEM + nab-PTX) improved PFS and OS compared to gemcitabine alone as a first-line regimen for metastatic PDAC (mPDAC) [2, 3].

In a continuum of care strategy, owing to the higher incidence of high-grade hematological and non-hematological treatment-related adverse events (AEs) with the FOLFIRINOX regimen, an intensive triplet association is usually reserved for fit PDAC patients in need of tumor shrinkage in the neoadjuvant setting (for borderline resectable or locally advanced tumors) or for symptom relief because of a high disease burden (in the metastatic setting) [4]. In the metastatic setting, a gemcitabine-based combination with nab-PTX is often the preferred 1st line regimen, particularly when taking into account funding restrictions in some countries. Overall, the treatment choice should be individualized for each patient and take performance status and co-morbidities into consideration [5].

After progression on a first-line gemcitabine-based treatment, different second-line regimens including combinations of folinic acid, 5-FU, and oxaliplatin (FOLFOX) or nanoliposomal irinotecan (NALIRI) and fluoropyrimidines, if not previously administered, demonstrated acceptable tolerability and a modest survival and clinical benefit [6–9]. The association of these chemotherapy backbones with targeted agents such as the PARP inhibitor veliparib [10] and the Janus kinase 1 (JAK1)/JAK2 inhibitor ruxolitinib [11] or pegylated recombinant human interleukin (IL)-10 (pegilodecakin) [12] have also been tested as alternative systemic treatments beyond standardly accepted second-line treatment options.

The aim of the present systematic review and network meta-analysis is to outline and aggregate the efficacy data of second-line treatments from these trials.

## Methods

### Objective

This study aimed to compare the efficacy of different 2nd line treatment regimens for patients with mPDAC after receiving 1st line gemcitabine-based systemic treatment. This systematic review and network meta-analysis (NMA) is reported according to the extension of the PRISMA (Preferred Reporting Items for Systematic Reviews and Meta-analyses) statement for reporting of systematic reviews incorporating network meta-analyses statements (Supplementary file) [13].

### Eligibility criteria

Phase 2–3 Randomized clinical trials (RCTs) were included if they: (1) included patients with metastatic PDAC progressing after gemcitabine-based first line chemotherapy, (2) reported PFS and/or OS outcome data, and (3) were published or presented in English. Trials of interest compared two or more active systemic treatments as second-line regimens until disease progression or unacceptable toxicity. Studies with unavailable full texts, with less than 30 patients randomized, including a pediatric population, and those that included non-adenocarcinoma histology, were not considered. Retrospective series, phase I studies, neoadjuvant studies in locally advanced PDAC, and trials comparing treatments that have not been approved by health authorities in both arms have been excluded as well. References of all papers included were scanned for additional studies of interest.

### Data source and search strategies

An electronic search of PubMed, Web of Science, Cochrane Library, and EMBASE was conducted from inception to May 2022. Searches were performed by using the following keywords and search string: ((“second line treatment” OR “previously treated” OR “pretreated”) AND (“pancreatic” OR “pancreas”) AND (“cancer” OR “adenocarcinoma”)) and were limited to RCTs. The search strategy, as well as the identification and review of records, was designed and performed by two researchers (A.P. and M.G.). In the case of duplicate publications, only the most complete, recent, and updated reports of the study were included.

### Data collection and risk of bias

The following data were recorded for each study: first author’s name, year of publication, name of the trial (if available), sample size, trial phase, intervention arms, and survival outcome results. The main outcomes of interest were OS and PFS, secondary endpoints were grade 3–4 hematological and non-hematological toxicities (nausea, vomiting, mucositis, diarrhea, and neurotoxicity). The risk of bias of each study was assessed according to The Cochrane Collaboration’s tool for assessing risk of bias. This tool assesses selection bias (random sequence generation and allocation concealment), performance bias, detection bias, attrition bias, reporting bias, and other sources of bias. The risk of bias from each study was assessed independently by two authors (F.P. and M.G.).

### Statistical analysis

NMA was performed under a Bayesian framework using the “gemtc” package (https://gemtc.drugis.org). Fixed effects and consistency models were also used. Non-informative priors were set, and posterior distributions were obtained using 4000 iterations after 1500 burns, and a thinning interval of 10. In the assessment for PFS and OS, contrast-based analyses were applied with estimated differences in the log HR and the standard error calculated from the published HR and CI. The relative treatment effects were presented as HR and 95% credible interval (CrI). The probability of each treatment in terms of survival outcomes was ranked according to HRs and posterior probabilities. For toxicities, arm-based analyses were performed to estimate the risk ratios (RRs) and 95% CrI from the available raw data presented in the selected manuscripts. We calculated the relative ranking of agents for each outcome as their surface under the cumulative ranking (SUCRA), which represents the percentage of efficacy or safety achieved by an agent compared with an imaginary agent that is always the best without uncertainty. A higher SUCRA score meant a higher ranking for efficacy outcomes. The convergence of the model evaluated the potential scale reduced factor (PSRF). If PSRF is close to 1, the convergence is considered favorable, the consistency of the homogeneity model would be considered reliable enough for analysis.

## Results

**Fig. 1:**
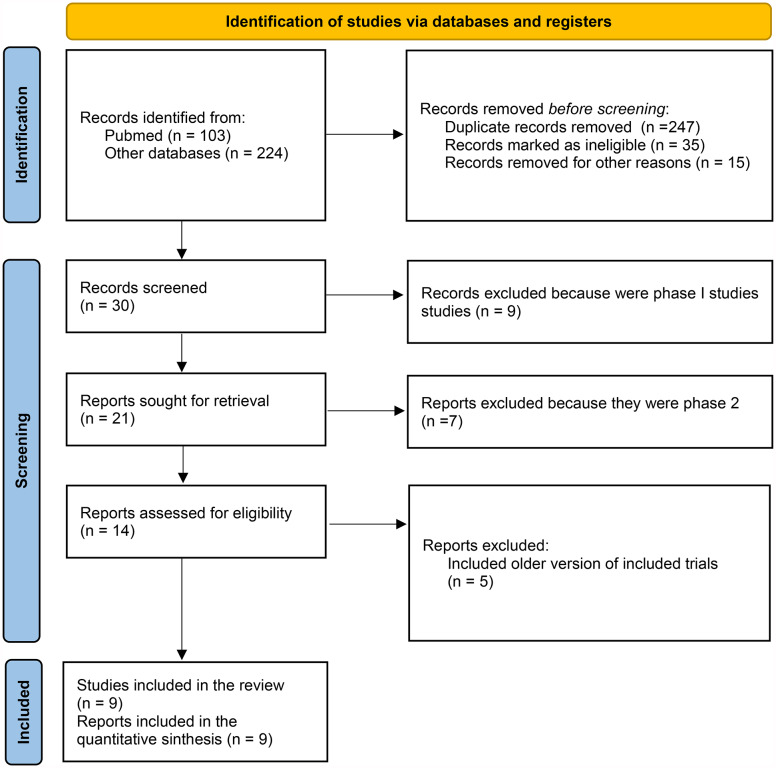
Flow diagram of the included studies

Among the 327 citations retrieved, 9 studies were included in the quantitative synthesis and network meta-analysis (NMA) (Fig. 1). The characteristics of the included studies are shown in Table 1. One study compared fluoropyrimidines alone or with folinic acid, 3 studies compared fluoropyrimidines + folinic acid with or without oxaliplatin, 2 studies compared fluoropyrimidines alone or with irinotecan (or liposomal irinotecan), and one study compared fluoropyrimidine-based therapies with or without investigational agents (capecitabine ± ruxolitinib, FOLFOX ± pegilodecakin and FOLFIRI ± veliparib). All studies had available data for the NMA of OS and PFS. The total number of enrolled patients was n = 2521.

**Table 1:** List of studies included in the quantitative analysis and Network Meta-Analysis. NS: not statistically significant according to preplanned statistical analysis

Author/year/ study name	Phase study/ Country	N° randomized patients	Study arms	Hematological toxicity G3-4 (exp arm) N/NF/PLT/Hb	Nonhematological toxicity G3-4 (exp arm) N/V/mucositis/diarrhoea/neurotoxicty	mPFS in months A/B	mOS in months A/B	Primary endpoint	Bias
Oettle/ 2014/ CONKO-003	III/Germany	168	Folinic acid + fluorouracil (FF) (arm A) vs. FF + oxaliplatin (OFF) arm B	NR/NR/1/3	3*/NR/0/3	2.0/2.9 h = 0.68 (SS)	3.3/5.9 h = 0.66 (SS)	OS	LOW
Wang-Gillam/ 2019/ NAPOLI-1	III/Global	417	5-FU/LV (arm A) vs. NALIRI + 5-FU/LV (arm B) vs. NALIRI (arm C)	15.4/NR/5.1/NR	NR/6/NR/9.4/NR	1.5/3.1 h = 0.57 (SS)	4.2/6.2 h = 0.75 (SS)	OS	LOW
Ohkawa/ 2015	IIR/Asia	271	S-1 (arm A) vs. SOX (S-1 + oxaliplatin) (arm B)	8/NR/10/8	6.6/2.9/1.5/5.1/2.9	2.8/3.0 h = 0.84 (NS)	6.9/7.4 h = 1.03 (NS)	PFS	SOME CONCERN
Ioka/ 2017	IIR/Asia	137	S-1 (arm A) vs. IRIS (S-1 + irinotecan) (arm B)	4.6/NR/4.7/15.6	6.3/3/1.6/3.1/NR	1.9/3.5 h = 0.77 (NS)	5.8/6.8 h = 0.75 (NS)	OS	SOME CONCERN
Hurwitz/ 2015/ RECAP	IIR/Western	127	Capecitabine (arm A) vs. capecitabine + Ruxolitinib (arm B)	0/NR/1.7/15.3	5/5/6.8/5/1.7	NA HR = 0.75 (NS)	4.3/4.5 h = 0.79 (NS)	OS	SOME CONCERN
Hecht/ 2021/ SEQUOIA	III/Global	567	FOLFOX (arm A) vs. FOLFOX + Pegilodecakin (arm B)	29.5*/NR/25/16	4/4/NR/4/2	2.1/2.1 h = 0.98 (NS)	5.8/6.3 h = 1.04 (NS)	OS	LOW
Gill/ 2016/ PANCREOX	III/Canada	108	mFOLFOX6 (arm A) vs. 5FU/LV (arm B)	32.7*/4.1/8.2/2	NR/4/NR/2/4	3.1/2.9 h = 0.90 (NS)	6.1/9.9 h = 1.68	OS	LOW
Chiorean/ 2021/ SWOG S1513	IIR/USA	123	FOLFIRI (arm A) vs. FOLFIRI + Veliparib (arm B)	34*/NR/NR/7	12/9/NR/11/NR	2.9/2.1 h = 1.39	6.5/5.4 h = 1.23 (NS)	OS	SOME CONCERN
Ioka/ 2018/ GRAPE	III/Asia	603	TAS-118 (S-1 + LV) (arm A) vs. S-1 (arm B)	1.7/NR/1.7/6	1.7/2/6.7/7.3/NR	3.9/2.8 h = 0.80	7.6/7.9 h = 0.98 (NS)	OS	LOW

Eight trials were two-arm design, and 1 trial was a three-arm design. All patients had received previous systemic therapy. All included trials were multicenter with an open-label randomized design. All studies except 1 had OS as the primary endpoint. Risk of bias was low in n = 5 studies, whereas some concern was raised in n = 4 smaller studies.

**Fig. 2:**
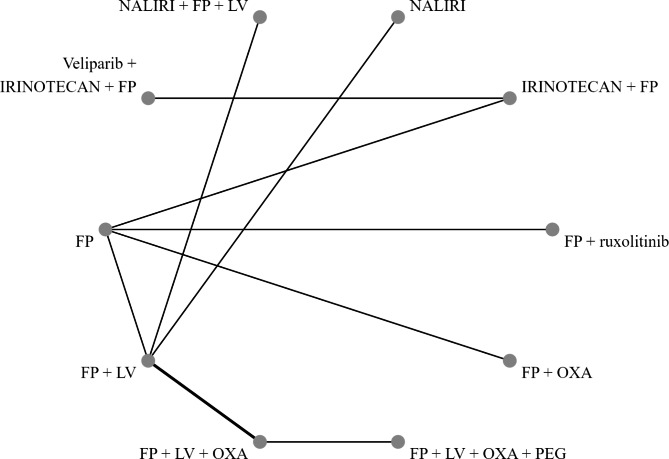
Network graph for OS. The width of the edge is proportional to the number of comparisons

**Fig. 3:**
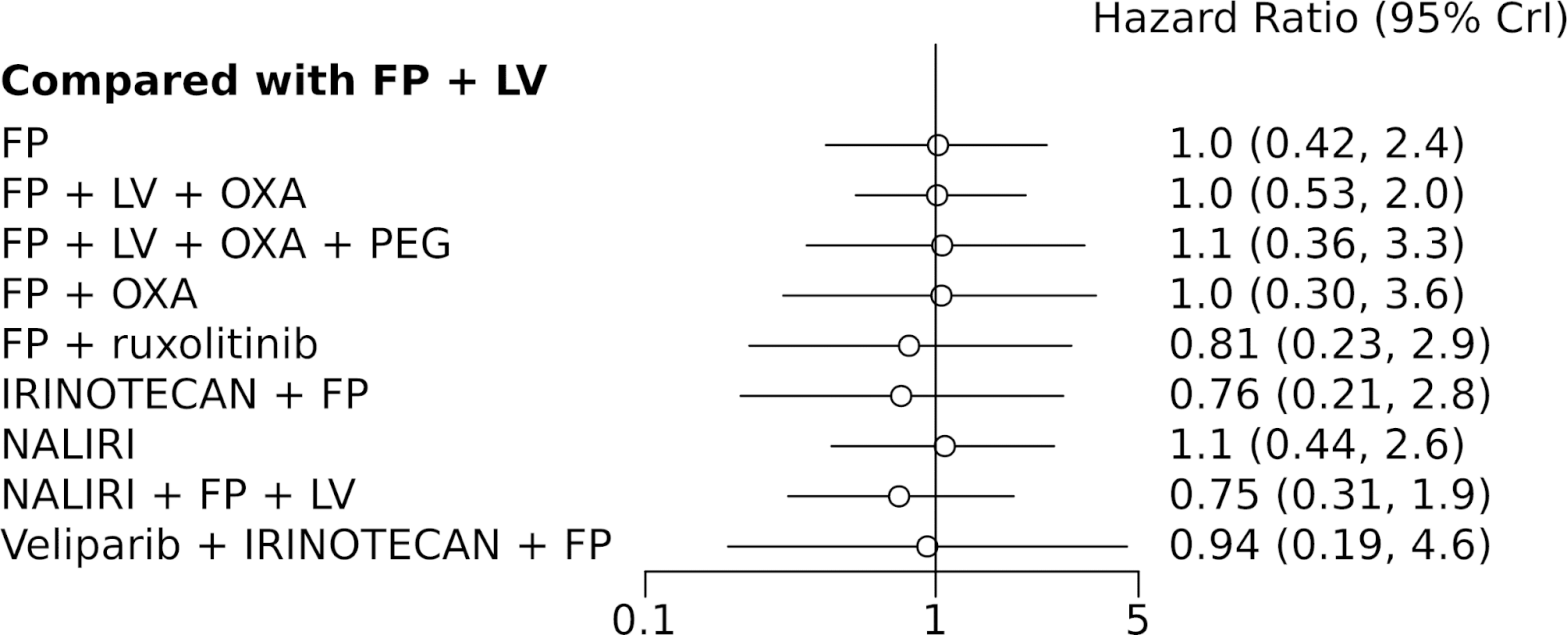
Bayesian comparisons for OS of various treatments vs. the referent standard arm

A NMA of 9 treatments was performed for OS. Compared with 5-FU + folinic acid, both irinotecan or NALIRI + fluoropyrimidines had a trend to better OS (HR = 0.76, 95%CI 0.21–2.75 and HR = 0.74, 95%CI 0.31–1.85). Fluoropyrimidines + folinic acid + oxaliplatin were no better than the combination without oxaliplatin (Table 2). Analysis of treatment ranking revealed that NALIRI + 5-FU + folinic acid had the highest likelihood of providing maximal OS benefit (SUCRA = 0.70). FOLFOX ± Pegilodecakin was ranked last (Table 3). The network graph and Bayesian comparisons for OS are shown in Figs. 2 and 3, respectively. The parameter PSRF value is 1.01, indicating the model’s convergence is good. Heterogeneity of comparisons was moderate (I^2^ = 55%).

**Table 2:** Comparison of the included interventions for OS: hazard ratio (95% CI). Each cell gives the effect of the column-defining intervention relative to the row-defining intervention

FP	*0.981 ( 0.414, 2.389)*	0.987 ( 0.339, 3.079)	1.035 ( 0.259, 4.316)	*1.031 ( 0.415, 2.497)*	*0.79 ( 0.310, 2.059)*	*0.74 ( 0.29, 1.90)*	1.058 ( 0.302, 3.693)	0.732 ( 0.213, 2.655)	0.928 ( 0.247, 3.376)
	FP + LV*	*1.012 ( 0.531, 2.040)*	1.053 ( 0.359, 3.256)	1.047 ( 0.299, 3.565)	0.81 ( 0.228, 2.935)	0.76 ( 0.21, 2.75)	*1.075 ( 0.438, 2.558)*	*0.74 (0.31, 1.85)*	0.936 ( 0.193, 4.560)
		FP + LV + OXA	*1.043 ( 0.429, 2.518)*	1.034 ( 0.245, 4.178)	0.79 ( 0.184, 3.309)	0.748 ( 0.173, 3.154)	1.061 ( 0.339, 3.146)	0.737 ( 0.238, 2.280)	0.920 ( 0.164, 5.169)
			FP + LV + OXA + PEG	0.993 ( 0.182, 5.175)	0.760 ( 0.138, 4.144)	0.721 ( 0.132, 3.904)	1.027 ( 0.239, 4.130)	0.713 ( 0.171, 2.998)	0.876 ( 0.129, 5.870)
				S1 + OXA^	0.772 ( 0.214, 2.822)	0.726 ( 0.197, 2.708)	1.022 ( 0.223, 4.678)	0.717 ( 0.156, 3.492)	0.891 ( 0.179, 4.455)
					CAPE + ruxolitinib	0.943 ( 0.239, 3.501)	1.336 ( 0.275, 6.423)	0.931 ( 0.195, 4.401)	1.167 ( 0.229, 5.819)
						IRINOTECAN + FP°	1.405 ( 0.305, 6.781)	0.987 ( 0.210, 4.744)	*1.225 ( 0.482, 3.074)*
							NALIRI	0.701 ( 0.201, 2.473)	0.881 ( 0.144, 5.266)
								NALIRI + FP + LV	1.246 ( 0.206, 7.622)
									Veliparib + IRINOTECAN + FP

*, include also S1 + leucovorin doublet; OFF, oxaliplatin + 5Fluorouracil + folinic acid; °, include FOLFIRI and S1 + irinotecan; ^, S1 + oxaliplatin; italics identifies direct comparisons

**Table 3:** The rank probability of the 9 regimens for OS: rank 1 represents the worst treatment and rank 10 represents the best

	Rank 1	Rank 2	Rank 3	Rank 4	Rank 5	Rank 6	Rank 7	Rank 8	Rank 9	Rank 10	SUCRA
FP	0.03	0.106	0.152	0.158	0.154	0.152	0.130	0.077	0.02	0.01	38%
FP + LV	0.02	0.078	0.141	0.182	0.169	0.152	0.126	0.083	0.03	0.01	39%
FP + LV + OXA	0.06	0.139	0.133	0.118	0.115	0.116	0.111	0.099	0.07	0.02	40%
FP + LV + OXA + PEG	0.19	0.131	0.092	0.085	0.081	0.077	0.082	0.081	0.08	0.09	41%
S1 + OXA	0.17	0.127	0.107	0.090	0.089	0.092	0.090	0.091	0.07	0.06	40%
FP + ruxolitinib	0.07	0.069	0.069	0.073	0.078	0.086	0.102	0.124	0.13	0.19	63%
IRINOTECAN + FP	0.02	0.068	0.068	0.070	0.072	0.081	0.099	0.142	0.20	0.17	69%
NALIRI	0.18	0.128	0.109	0.096	0.094	0.088	0.084	0.078	0.07	0.06	38%
NALIRI + FP + LV	0.04	0.052	0.053	0.059	0.081	0.087	0.096	0.129	0.15	**0.24**	**70%**
Veliparib + IRINOTECAN + FP	0.18	0.103	0.075	0.068	0.067	0.069	0.078	0.096	0.12	0.13	50%

*, include also S1 + leucovorin doublet; °, include FOLFIRI and S1 + irinotecan

**Fig. 4:**
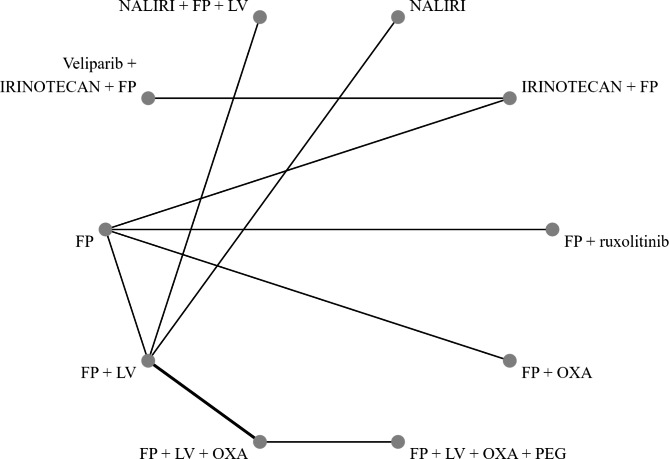
Network graph for PFS. The width of the edge is proportional to the number of comparisons

**Fig. 5:**
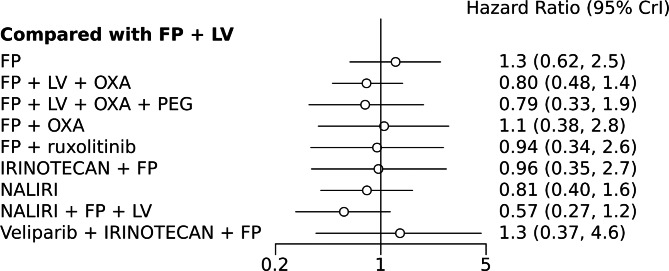
Bayesian comparisons for PFS of various treatments vs. the referent standard arm

When PFS NMA was performed, NALIRI + 5-FU and folinic acid was ranked as the best-performing treatment compared to all other regimens (SUCRA = 0.91; Table 4). Conversely, FOLFIRI + veliparib was ranked last (Table 5). The network graph and Bayesian comparisons for PFS are shown in Figs. 4 and 5, respectively. The parameter PSRF value is 1.02, indicating the model’s convergence is good. Heterogeneity was moderate (I^2^ = 55%).

**Table 4:** Comparison of the included interventions for PFS: hazard ratio (95% CrI). Each cell gives the effect of the column-defining intervention relative to the row-defining intervention

FP	*0.798 ( 0.401, 1.601)*	0.640 ( 0.274, 1.556)	0.630 ( 0.210, 1.927)	*0.841 ( 0.409, 1.705)*	*0.755 ( 0.355, 1.558)*	*0.773 ( 0.362, 1.635)*	0.646 ( 0.240, 1.713)	0.454 ( 0.166, 1.239)	1.076 ( 0.371, 3.027)
	FP + LV*	*0.804 ( 0.478, 1.395)*	0.787 ( 0.334, 1.921)	1.051 ( 0.385, 2.823)	0.944 ( 0.343, 2.603)	0.964 ( 0.347, 2.719)	*0.808 ( 0.400, 1.623)*	*0.56 ( 0.27, 1.15)*	1.342 ( 0.372, 4.635)
		FP + LV + OXA	*0.981 ( 0.483, 1.960)*	1.314 ( 0.412, 4.008)	1.174 ( 0.365, 3.601)	1.198 ( 0.365, 3.849)	1.009 ( 0.403, 2.386)	0.709 ( 0.285, 1.708)	1.665 ( 0.407, 6.489)
			FP + LV + OXA + PEG	1.341 ( 0.351, 5.044)	1.202 ( 0.314, 4.528)	1.223 ( 0.311, 4.676)	1.031 ( 0.335, 3.043)	0.727 ( 0.228, 2.197)	1.700 ( 0.358, 7.645)
				S1 + OXA	0.894 ( 0.319, 2.506)	0.916 ( 0.327, 2.599)	0.769 ( 0.230, 2.555)	0.538 ( 0.158, 1.820)	1.270 ( 0.358, 4.648)
					CAPE + ruxolitinib	1.023 ( 0.349, 2.925)	0.864 ( 0.260, 2.898)	0.602 ( 0.178, 2.063)	1.426 ( 0.387, 5.229)
						IRINOTECAN + FP°	0.835 ( 0.249, 2.858)	0.584 ( 0.167, 2.079)	*1.394 ( 0.647, 2.901)*
							NALIRI	0.701 ( 0.256, 1.928)	1.654 ( 0.387, 6.851)
								NALIRI + FP + LV	2.379 ( 0.540, 10.164)
									Veliparib + IRINOTECAN + FP

*, include also S1 + leucovorin doublet; °, include FOLFIRI and S1 + irinotecan;

**Table 5:** The rank probability of the 9 regimens for PFS: rank 1 represents the worst treatment and rank 10 represents the best

	Rank 1	Rank 2	Rank 3	Rank 4	Rank 5	Rank 6	Rank 7	Rank 8	Rank 9	Rank 10	SUCRA
FP	0.18	0.309	0.235	0.130	0.066	0.039	0.023	0.010	0.01	0.01	9%
FP + LV	0.03	0.083	0.140	0.188	0.210	0.200	0.097	0.037	0.01	0.01	36%
FP + LV + OXA	0.01	0.034	0.048	0.063	0.093	0.130	0.190	0.233	0.15	0.03	73%
FP + LV + OXA + PEG	0.05	0.049	0.047	0.058	0.077	0.097	0.130	0.165	0.19	0.12	70%
S1 + OXA	0.11	0.129	0.150	0.149	0.128	0.095	0.080	0.069	0.05	0.03	33%
CAPE + ruxolitinib	0.07	0.087	0.104	0.119	0.126	0.115	0.102	0.099	0.10	0.07	48%
IRINOTECAN + FP	0.02	0.128	0.120	0.139	0.123	0.115	0.102	0.097	0.09	0.05	45%
NALIRI	0.04	0.045	0.050	0.064	0.092	0.123	0.172	0.144	0.18	0.08	70%
NALIRI + FP + LV	0.01	0.010	0.013	0.017	0.025	0.040	0.062	0.101	0.16	**0.55**	**91%**
Veliparib + IRINOTECAN + FP	0.45	0.127	0.093	0.073	0.060	0.047	0.042	0.044	0.03	0.02	19%

Neutropenia (RR 2.9, 95%CI 2.4–3.6; P < 0.01) and thrombocytopenia (RR 2.2, 95%CI 1.7-3; P < 0.01) G3-4 were significantly worst in FOLFOX-based regimens (SUCRA = 0.84). NALIRI-based and FOLFIRI-based arms were associated with an increased risk of diarrhea (RR 2.7, 95%CI 1.9–3.7; P < 0.01) (SUCRA = 0.76).

### Sensitivity analysis for OS

Five phase 3 trials were included. Results did not show relevant deviations compared with the original NMA, but showed a higher probability of ranking NALIRI + 5-FU + folinic acid compared to 5-FU + folinic acid alone (SUCRA = 0.96) better for OS benefit. Analysis according to country of origin was not feasible because all treatments were not linked each other.

## Discussion

Second-line chemotherapy in mPDAC is usually considered for patients who retain a good performance status after progression on first-line treatment. For example, in the MPACT trial, 68% of patients after first-line gem + nab-PXT and 75% of patients after gemcitabine single-agent therapy, had a Karnofsky performance status of 90–100 [14]. Overall, less than 50% of patients included in the phase-III trials MPACT and PRODIGE received second-line treatment. Specifically, 40 and 44% of patients in the MPACT trial (treated with gem + nab-PTX and gemcitabine, respectively) [3] versus 46.8 and 49.7% treated with FOLFIRINOX and gemcitabine in the PRODIGE trial, respectively, received chemotherapy after progression on first-line treatment [2]. Sarcopenia and hypoalbuminemia at baseline have been reported as negative predictive factors for receiving second-line chemotherapy after first-line treatment with GEM + nab-PXT.

Currently, there is no standard second-line treatment recommended for mPDAC, and treatment choice is based on many factors such as the patient’s clinical condition, previous treatment, possible pre-existing toxicities, and regulatory issues that preclude prescription of specific drugs such as nab-PTX in second and further lines in some countries. After first-line gemcitabine-based treatment, multiple regimens including NALIRI + 5-FU and folinic acid, FOLFIRINOX, 5-FU-based oxaliplatin doublets (e.g., OFF, FOLFOX, or XELOX), or 5-FU-based monotherapy (FL, capecitabine, or S-1) are listed in major guidelines as appropriate therapies [15]. Recently, a retrospective, multicenter, real-world study evaluated the efficacy and safety of adding nab-PTX to gemcitabine after FOLFIRINOX treatment failure. This combination treatment was associated with a significantly better disease control rate (DCR), PFS, and OS than gemcitabine alone in patients with mPDAC but at the expense of a higher rate of grade 3 and 4 toxicities [16]. Similarly, second line FOLFIRINOX has been evaluated in many retrospective series after progression on gemcitabine-based first-line treatment. A real-world Italian study compared second-line FOLFIRINOX to FOLFOX and FOLFIRI. Compared with FOLFIRI, FOLFIRINOX was reported to have significantly better survival outcomes in terms of both median PFS and median OS from the start of second-line treatment (OS2). In contrast, no significant differences were observed between the FOLFIRINOX and FOLFOX groups in terms of PFS and OS2 [17].

In our NMA, we only included phase II and III randomized trials. None of these studies evaluated FOLFIRINOX as second-line treatment. Two randomized phase III trials have evaluated the addition of oxaliplatin to 5-FU and folinic acid, with conflicting results. Indeed, CONKO-003 showed a statistically significant improvement both in terms of PFS and OS with the combination treatment [6], while in the PANCREOX study, the use of oxaliplatin was detrimental with poorer OS compared to 5-FU and folinic acid monotherapy [18, 19]. Therefore, whilst the addition of oxaliplatin to 5-FU and folinic acid remains an accepted 2nd line treatment option with its inclusion in guidelines, the evidence base supporting its use has not been consistently replicated. NALIRI in combination with 5-FU and folinic treatment demonstrated the greatest potential for achieving the highest PFS results (91% likelihood of being the most effective regimen). Similarly, irinotecan-based regimens (NALIRI or FOLFIRI) also ranked highly in terms of the OS analysis, despite being more toxic in terms of diarrhea.

Recently, a multicenter retrospective study by the Korean Cancer Study Group compared second line NALIRI + 5-FU + folinic acid to second line FOLFIRINOX. The analysis found no significant difference in the objective response rate, PFS, and OS between the two treatments. Interestingly, patients treated with FOLFIRINOX had better OS if they were < 70 years old, whereas patients treated with NALIRI + 5-FU + folinic acid had better PFS and OS if they were ≥ 70 years old [20]. This suggests that NALIRI + 5-FU + folinic acid might represent a good treatment option for elderly, pretreated mPDAC patients, with FOLFIRINOX being reserved for highly selected, mostly younger, fitter patients. However, these findings would need to be confirmed in a prospective study.

The first results of the HR-IRI-APC phase III trial were presented at the ESMO 2022 meeting. This study enrolled 298 patients with mPDAC who had previously failed gemcitabine-based therapies. Patients were randomized to receive either HR070803, a liposomal formulation of irinotecan plus 5-FU and folinic acid, or placebo plus 5-FU and folinic acid. The study met is primary endpoint of OS, with 7.39 months in the treatment arm and 4.99 months in the placebo arm ([HR] = 0.63; 96.4% CI, 0.48 to 0.84; p = 0.0019). The median PFS was 4.21 months in the investigational arm compared with 1.48 months in the chemotherapy plus placebo arm ([HR] = 0.36; 95% CI, 0.27 to 0.48; p < 0.0001). The ORR was 12.75% (95% CI, 7.86 to 19.20) in the treatment arm and 0.67% (95% CI, 0.02 to 3.68) in the placebo arm (p < 0.0001). Although these results support the efficacy of the new irinotecan formulations in association with 5-FU in the second line setting of mPDAC, the study was conducted in an Asian population only. Therefore, evaluation of HR070803 in the Western population is warranted to assess the applicability of these findings in other populations [21].

The most recent NMA of second line treatments included 8 studies and was conducted in 2018 by Citterio et al. Their results demonstrated that FOLFIRI-based combinations were the best in terms of both OS and PFS for patients not previously treated with these drugs. Within the present NMA, we considered 2 more studies, including the NALIRI + 5FU doublet. No other NMAs compared salvage treatments for PDAC in NMA in the last 5 years.

This study had several limitations. First, no RCTs compared the efficacy of FOLFIRINOX as second-line treatment. Second, the heterogeneity of patients included in terms of race and first-line therapies (single agents vs. chemotherapy doublets regimens) may have limited the applicability of our results. Third, the inclusion of small phase II studies with limited follow-up was another limitation of this NMA. Finally, a possible recommendation as a new standard cannot be formulated because, in older studies, first-line therapies were mainly represented by gemcitabine alone or with platinum agents and not by the established first-line therapies.

## Conclusions

The choice of optimal second-line treatment in patients with mPDAC remains challenging due to the lack of randomized trials comparing combination chemotherapy regimens. Our NMA revealed that irinotecan-based regimens based (such as NALIRI or FOLFIRI) may be the preferred options for second-line treatment with regards to survival outcomes, particularly PFS. Further evidence from prospective trials is needed to determine the best treatment option for this group of patients.

## Electronic supplementary material

Below is the link to the electronic supplementary material.

Supplementary Material 1

## Data Availability

Datasets and materials used during the present study are available from the corresponding author upon reasonable request.
